# The null hypothesis significance test and the dichotomization of the p-value: *Errare Humanum Est*

**DOI:** 10.17843/rpmesp.2024.414.14285.

**Published:** 2024-11-26

**Authors:** Edward Mezones-Holguín, Ali Al-kassab-Córdova, Percy Soto-Becerra, Sonia Hernández-Díaz, Jay S. Kaufman

**Affiliations:** 1 Centro de Excelencia en Investigaciones Económicas y Sociales en Salud, Universidad San Ignacio de Loyola, Lima, Peru. Universidad San Ignacio de Loyola Centro de Excelencia en Investigaciones Económicas y Sociales en Salud Universidad San Ignacio de Loyola Lima Peru; 2 Vicerrectorado de Investigación, Universidad Continental, Huancayo, Peru. Vicerrectorado de Investigación Universidad Continental Huancayo Peru; 3 Department of Epidemiology, Harvard T. H. Chan School of Public Health, Boston, Massachusetts, EE.UU. Department of Epidemiology Harvard T. H. Chan School of Public Health Boston Massachusetts EE.UU.; 4 Department of Epidemiology, Biostatistics, & Occupational Health, McGill University, Montreal, Canada. McGill University Department of Epidemiology, Biostatistics, & Occupational Health McGill University Montreal Canada

**Keywords:** Statistical Analysis, Hypothesis-Testing, Biostatistics, Epidemiology and Biostatistics, statistics & numerical data

## Abstract

Decision-making in healthcare is complex and needs to be based on the best scientific evidence. In this process, information derived from statistical analysis of data is crucial, which can be developed from either frequentist or Bayesian perspectives. When it comes to the frequentist field, the null hypothesis significance test (NHST) and its p-value is one of the most widely used techniques in different disciplines. However, NHST has been subjected to questioning from different academic points of view, which has led to it being considered as one of the causes of the so-called replicability crisis in science. In this review article, we provide a brief historical account of its development, summarize the underlying methods, describe some controversies and limitations, address misuse and misinterpretation, and finally give some scopes and reflections in the context of biomedical research.

## INTRODUCTION

The use of scientific evidence is crucial in the context of health decision-making. Scientific research generally follows two main approaches: the empirical-inductive approach focused on generalization from specific observations and the hypothetico-deductive approach based on the evaluation of the validity of a specific hypothesis ^1^. In biomedical research, data analysis is a challenge for both observational and experimental studies, where validity and precision are key; however, these properties are threatened by systematic and random errors ^2^. In practice, one of the challenges we face is to infer findings in the population of interest based on a sample, through a formal procedure of statistical inference with mathematical models that seek to reflect a usually complex phenomenon ^3^. In statistical analysis there are two main trends: frequentist and Bayesian; ^4^ so it is critical to understand the differences and similarities between the two approaches in order to adequately select and plan the study design, sampling techniques and data analysis.

Among the many frequentist techniques of statistical inference, the most commonly used are the null hypothesis significance test (NHFT) and confidence intervals (CI) ^5^^-^^7^. In both cases, we seek to answer questions about sample-based populations, with the possibility of calculating multiplicative or additive measures of association (i.e., hazard ratio or risk difference, respectively), which in the absence of systematic errors and under certain assumptions, could be interpreted as causal (i.e., of effect) ^2^^,^^8^. Specifically, the NHFT tests hypotheses in a given population of interest by estimating the p-value ^6^^,^^9^.

Although the NHFT is the dominant approach in several areas of knowledge, it has been subject to controversy and criticism since its formulation ^5^^,^^7^^,^^10^^-^^12^. The p-value is the probability of obtaining a result equal to the observed one, or a more extreme one, if the null hypothesis is true; and although it contains useful information, it does not represent the magnitude of the evaluated association ^6^^,^^7^^,^^9^^,^^13^. Some authors have stated that the NHFT is one of the most abused and misinterpreted statistical analysis techniques ^7^^,^^10^^,^^11^, a situation that has contributed to the so-called replicability crisis in scientific research ^14^^-^^17^. Subsequently, knowing more about this metric in the biomedical research setting is relevant.

Although NHFT is used in many types of biomedical studies, in this article we address its use in primary studies (analysis of data from the primary units of analysis; i.e. observational studies and experimental studies) and mainly for association testing (since it can be used in other settings, such as in the case of comparison of distributions). First, we introduce the methods and give a brief historical account. Then, we summarize some controversies and limitations. Afterwards, we note some misuses and misinterpretations. Finally, we succinctly point out some reflections on the above problems.

## HISTORICAL AND CONCEPTUAL BASES

The NHFT is used to reject or not a null hypothesis based on the role that random sampling error may play ^2^^,^^18^. The NHFT evolved from the combination of two divergent philosophical orientations developed simultaneously by Ronald Fisher, and by Jerzy Neyman and Egon Pearson ^9^^,^^13^^,^^19^.

### The significance test or significance probability

Published in 1925 by Ronald Fisher, this technique evaluated whether the result is significant by means of the significance probability (SP), a measure of the consistency between the data and the null hypothesis with values from 0 to 100%, where the lower the value, the greater the consistency. The SP was proposed as an inferential tool that sought to move away from the subjectivism of the Bayesian orientation. Fisher considered that this tool should be combined with other sources of information and if a threshold is used, it should be flexible and vary according to the accumulated knowledge on the research question ^13^^,^^19^^,^^20^.

### The hypothesis test

In 1933, Jerzy Neyman and Egon Pearson proposed the inclusion of alternative hypotheses (initially formulated in 1928) and a theoretical approach that involved defining and considering type 1 and type 2 random errors. They sought to estimate a minimum relevant effect based on the quantification of the magnitude of the random error and its long-term adjustment with the use of critical regions in order to define the rejection or non-rejection of a hypothesis, under the assumption that robust conclusions could not be established from a single study ^13^^,^^19^^,^^20^.

After years of constant criticism of the thinking between both schools, around 1940, other researchers -among them Lindquist- created a system that gathered both approaches; they called it: hypothesis test based on the p-value, statistical significance test or significance test of the null hypothesis ^13^. In this proposal they excluded some points related to that formulated by Fisher (regarding the paraphrase of the incorporation of accumulated knowledge) and by Neyman and Pearson (which allows interpreting as limited the conclusion derived from a single experiment) ^9^^,^^13^^,^^19^^,^^21^. Precisely the conceptualization of the NHFT from two approaches with differentiated methods and terminologies have contributed to the development of controversies in the academy ^9^^,^^13^^,^^21^.

**Figure 1:**
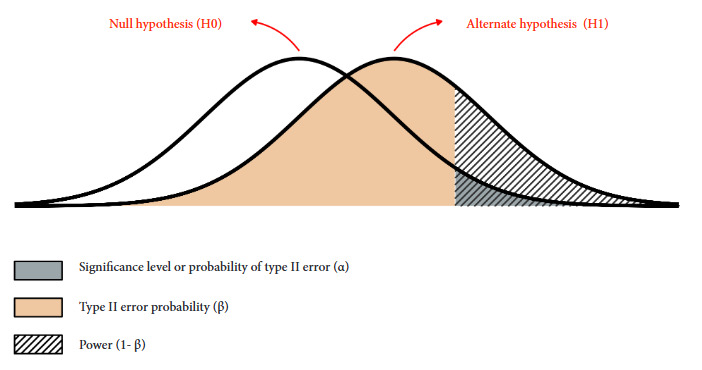
Probability distribution of randomly obtained test sample statistics in null or true alternate hypothesis scenarios.

To understand the NHFT, we must know about random sampling errors: type 1 error refers to the false positive of rejecting a true null hypothesis; while type 2 error corresponds to the false negative of not rejecting the null hypothesis when it is false. Under the frequentist perspective, we can control this error by prefixing the probability of occurrence of these errors if infinite possible samples of the same size were taken. The probability of type l error (α) prefixed in the design is known as significance and its complement (1-α) is known as confidence. Whereas, the probability of type 2 error is denoted as β and its complement (1-β) as statistical power ^2^^,^^18^. To illustrate these concepts, in Figure 1 we present the probability distribution of the test sample statistics that we would randomly obtain in two possible scenarios: if the null hypothesis is true or if the alternative hypothesis is true for a given effect size.

We usually establish both probabilities during the sample size calculation, where we predefine the α and after the execution of the test, we estimate the p-value; it is according to the contrast between the p-value and the α, that we reject or not the null hypothesis and thus define the rejection based on the magnitude of the incompatibility between the observed data and the null hypothesis ^6^^,^^9^^,^^13^. In a context where they are often used interchangeably, it is crucial that we differentiate the p-value from the NHFT; in general, the NHFT corresponds to the testing process and is its main indicator ^2^^,^^9^^,^^18^.

The NHFT corresponds to the specification of a null hypothesis about population parameters, where we state the non-existence of association or differences expressed on an additive or multiplicative scale in a statistical model. In addition, we consider alternative hypotheses (two-tailed hypotheses) that present one-tailed or two-tailed association or differences; the latter is the most frequently used and tests the existence of association or differences independently of their direction; on the other hand, , the association or difference is tested in favor of one of the directions in the one-tailed hypothesis ^(^^6^^,^^13^^,^^18^.

During the NHFT process, we calculate the observed test statistic for a specific model and from the expected distribution of the statistic if the null hypothesis were true, we estimate a p-value, which turns out to be the probability of obtaining a test statistic equal to or larger than that observed in our sample if we were to repeat sampling an infinite number of times under a true null hypothesis. Thus, the p-value tells us that when there is no effect (null effect), no association, or no difference, it is possible to see nonzero sample estimates of effect (i.e., values such as 1, 2, or 10 mm Hg blood pressure differences) simply by chance and quantifies how likely it is to observe these differences or more extreme differences if there really were no such differences in the population. Thus, a very small p-value implies that, although possible, it is very unlikely to have obtained a test statistic equal to or larger than that observed from a true null hypothesis. In this scenario, to assume that the null hypothesis is true would be to recognize that the improbable is more likely to happen, so the most reasonable and usual thing to do is to use the infrequent event rule, where we consider that the null hypothesis cannot be true and reject it, thus opting for the alternative ^6^^,^^9^^,^^18^^,^^22^. Additionally, not being able to reject the null hypothesis is not equivalent to confirming it and this is a critical point in the construction of the conclusions ^7^^,^^13^^,^^18^.

**Figure 2:**
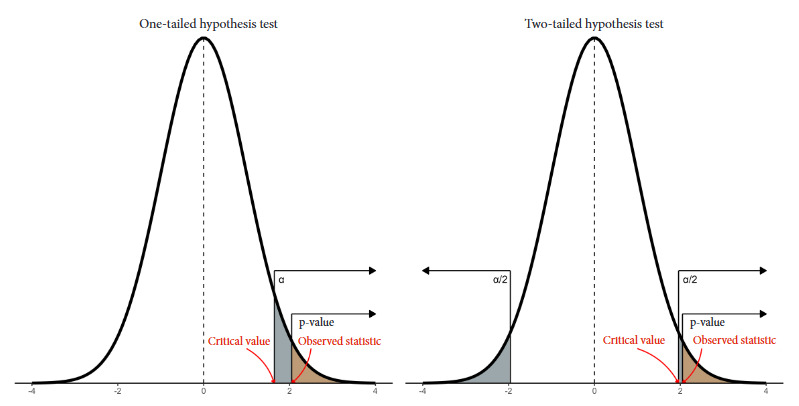
Probability curve under the null hypothesis of all possible values of the one-tailed and two-tailed test statistic.

In Figure 2 we show the curve denoting the probability under the null hypothesis of all possible values of the test statistic of a particular study, both one-tailed and two-tailed. On the right-hand side we observe the area corresponding to α and the p-value. Although they are similar, the key difference lies in the fact that α is pre-specified by us, while the p-value is calculated from the observed data in the study.

We re-emphasize that the p-value only takes into account random error, so its nominal interpretation should be taken exclusively in the absence of other errors in the study, such as selection bias, measurement bias, confounding, or error in the specification of the model ^2^^,^^7^^,^^22^. In this sense, the lack of randomization in observational studies or the violation of randomization in experimental studies brings with it a greater problem than the NHFT, as it affects the validity of the results ^2^^,^^5^^,^^22^^,^^23^.

## CONTROVERSIES AND LIMITATIONS

In this section we address some critical issues regarding the NHFT and p-value, although we present them independently, in practice these may overlap and interact.

### Problems in the design

Beyond the controversy of its creation from two opposing statistical approaches, it has been argued that the NHFT and the p-value have problems in their very conception. The NHFT is based on assessing the probability of obtaining the observed data under the assumption that a specific null hypothesis is true [Pr (observed data | true H_0_) ]. However, what we are really interested in is to calculate the probability that the null hypothesis is true given the evidence collected [Pr (H_0_true|data) ]. Thus, to make this logical leap, we perform a procedure recognized in epistemology as inverse deduction or reduction method, when the observed finding is improbable, to obtain a conclusion about the probability of the null hypothesis given the conclusion found in the data, for which we need to make several assumptions ^24^^-^^26^. It is also argued that, under the frequentist approach, it is difficult to cross the logical gap from the probability of the finding and more extreme findings, given a certain null hypothesis, to a decision on whether to accept or reject that hypothesis ^9^^,^^12^^,^^14^^,^^17^^,^^25^^,^^26^. Consequently, the interpretation of results based on the NHFT and p-value requires us to be fully aware of the assumptions underlying the process.

### Sample size dependence and discordance with effect size

Since NHFT require an *a priori* specification of the probability of type 1 error, this has a direct implication on the calculated sample size and on the p-value accepted for the definition of significance ^6^^,^^13^^,^^18^. In large samples, even if the effect was minimal, the p-value could be extremely small, which facilitates the rejection of the null hypothesis, regardless of the prefixed type 1 error size. Thus, the p-value could be as small as the sample size allows, making the analysis susceptible to manipulation ^10^^-^^12^. For example, in the case of analyses with massive data (big-data), the p-value estimate could practically define any association as statistically significant ^9^^,^^11^^,^^23^.

In this sense, although the p-value is a function of sample size and provides us with the probability of observing the estimated test statistic, this statistic does not provide us with information on the magnitude of the observed effect. Since it is a composite metric that is highly dependent on the sample size, it implies that small differences in the effect in a sufficiently large number of observations may be statistically significant; conversely, larger differences in effect in a small number of observations may not be significant ^7^^,^^10^^,^^23^^,^^27^^,^^28^. Thus, it has been estimated in clinical trials that statistical significance tends to seriously overestimate the treatment effect and that some non-significant results correspond to important effects ^29^^,^^30^.

### Non-correspondence with clinical significance

Statistical significance is not equivalent to scientific, human, economic or clinical importance ^23^^,^^29^^,^^30^^,^^33^^,^^34^. We reiterate that the formal definition of p-value is a mathematical concept expressed as a probability conditional on nullity, so it is feasible that there are statistically significant but clinically irrelevant differences, or the reverse ^6^^,^^7^. It is recognized that clinical differences are more important than statistical differences, thus, the evaluation of the p-value is secondary in the grading of the quality of evidence and is not a measure of evidence in itself ^23^^,^^35^.

**Figure 3:**
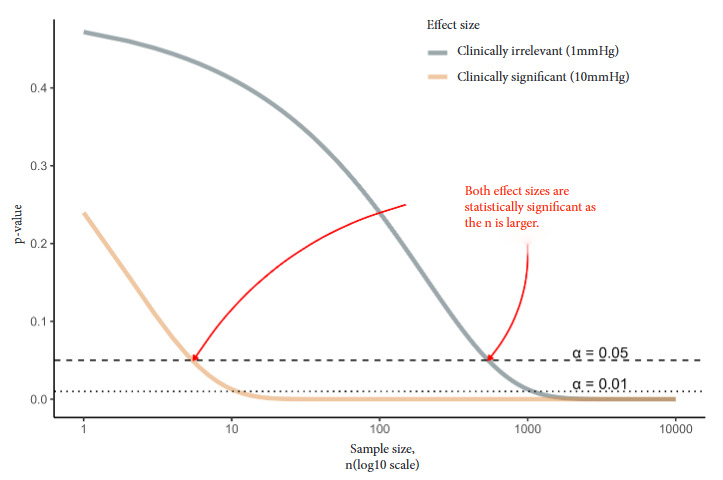
Clinically irrelevant or statistically significant scenarios for example in changes in arterial hypertension.

To illustrate the above, in Figure 3 we show an example with blood pressure for a clinically irrelevant scenario (1 mm Hg reduction) and a clinically significant scenario (10 mm Hg reduction). For the same effect size value and prespecified α, the p-value changes as the sample size increases (both effect sizes are statistically significant as the sample size increases); however, the clinical implications are different.

### Arbitrary categorization/dichotomization

The p-value is a continuous numerical measure with values between 0 and 1; however, it is most often interpreted in the binary form: statistically significant and not statistically significant, based on an arbitrary threshold that usually corresponds to 0.05 in some literature but some reports consider a p-value of less than 0.01 as highly significant ^9^^-^^11^. It has been estimated that in the biomedical literature (based on articles indexed in Medline) 96% of abstracts and full-text articles report p-values of 0.05 or less ^5^. Although it has been further classified in other disciplines as: highly significant, marginally significant and statistically non-significant; with cut-off points at 0.01 and 0.1^6^. We must emphasize that categorization leads to loss of relevant information on the parameter of interest ^7^^,^^11^^,^^36^. Based on this, some researchers argue that if we use the p-value it should be presented in its continuous nature and should always be considered under a specific context ^29^^,^^37^.

### Poor replicability, the curse of the winner, and vices derived from the search for statistical significance

The reliability of the NHFT and the p-value has been called into question, due to the high frequency of statistically significant positive scientific findings, which are contradicted in subsequent studies or in repeated experiments ^14^^-^^16^^,^^38^^-^^40^. It is precisely the combination of sample size dependence and arbitrary dichotomization that - among other factors - has brought criticism for poor replicability and high false positive rate ^28^^,^^30^^,^^33^^,^^39^.

It has been described that, under the 0.05 threshold for the p-value, there is a risk of false positive findings of 13% in clinical trials published in journals indexed in Pubmed-Medline ^41^, and discordances have also been found in other series of clinical trials ^42^. In addition, based on data from the Open Science Collaboration, a correlation coefficient of 0.004 (low) was calculated between the p-values obtained from the original study cohort and those estimated from the replication cohorts ^28^. Nine out of ten clinical trials do not reach a statistical power of 80% (median:13%) and most could not address the effect of the intervention ^31^. This implies that, if evidence is produced with statistically significant results with insufficient power, the effect size could be exaggerated, thus not being replicable in future studies and leading to misinterpretations. This phenomenon is known as the winner’s curse and was coined from the idea of an auction where the true value of the auctioned item is being guessed. The winner of the auction is the one who pays the highest price to compete with other potential buyers bidding on the item. Although the average of all bids is unbiased, the price paid at the end by the winner is definitely the one that overestimates the true value of the item by the greatest magnitude. Thus, the researcher with the statistically significant result is like the winner of the auction: he or she will surely be the one who is furthest away from the true value, often overestimating it ^31^^,^^32^. Thus, the p-value *curses* the researcher with inflated but statistically significant effect estimates.

In addition, there are several bad research practices related to the search for finding and reporting statistically significant results, among which we find multiple comparison, cherry-picking, fishing expeditions, data dredging, P-hacking and publication bias ^43^. When we conduct multiple statistical tests within the same study, the probability of finding at least one statistically significant result by chance without a real association or effect increases; given that a typical article contains dozens of tests, a percentage of them could be statistically significant, which when highlighted lead to a replication error ^33^^,^^44^^-^^46^. Likewise, manipulation in the search for significant results in selective data analysis or the use of multiple tests until a desired outcome is achieved ^47^. Taken together, these practices result in an increased likelihood of type 1 error and misleading conclusions ^22^.

In the face of the growing wave of criticism, the American Statistical Association (ASA) made a statement with six key statements about the p-value, which we show in Table 1 ^30^. Although it has had positive and negative reactions in academia ^48^^,^^49^; in our opinion, the ASA position constitutes a valid effort in the attempt to redirect scientific and academic practice.

**Table 1:** American Statistical Association principles of p-value (Taken from: Wasserstein &Lazar) ^30^.

Principles
The p-values can indicate the level of inconsistency between the observed data with respect to that pre-specified in a statistical model.
p-values do not measure the probability that the hypothesis under study is true or the probability that the information generated from the data is produced by chance alone.
Scientific conclusions, as well as commercial, clinical or political decisions should not be based solely on the fact that the p-value passes a specific threshold.
A proper inference process requires full reporting and transparency.
The p-value or statistical significance is not a measure of effect size or significance of the result.
The p-value by itself does not provide a good measure of evidence regarding a model or hypothesis.

## MISUSE AND MISINTERPRETATIONS

The questioning of the NHFT and the p-value extends to its incorrect use and erroneous interpretations, which constitutes one of the most serious problems affecting the quality of scientific research in many areas ^12^^,^^15^^,^^16^^,^^37^. The complexity of its interpretation added to the ease of calculating it in statistical packages may explain the excessive and inappropriate use of the p-value ^22^^,^^36^^,^^38^. Although clinicians and decision-makers often have high confidence in the estimation of the p-value, its interpretation can be counterintuitive and generally incorrect ^30^^,^^34^^,^^35^. Misinterpretation problems have even been reported in professionals with postgraduate training in statistics and epidemiology ^50^. Although there are multiple forms of misinterpretation and each one requires a particular analysis, in Table 2 we present the most common ones adapted from Greenland *et al.*^7^.

**Table 2:** Common misinterpretations regarding the significance test of the null hypothesis and the p-value (Adapted from Greenland *et al*.) ^7^

Erroneous Interpretations
The p-value is the probability that the null hypothesis is true.
The p-value is the probability that chance alone produces the observed association.
A statistically significant result (p≤0.05) means that the null hypothesis is false or should be rejected.
A non-statistically significant result (p>0.05) means that the null hypothesis is true and should not be rejected.
A large p-value is evidence in favor of the null hypothesis.
A p-value greater than 0.05 means that a non-effect was found or that the absence of an effect was demonstrated.
Statistical significance scientifically indicates that an important relationship has been detected.
The absence of statistical significance indicates that the effect size is small.
The p-value is the probability that our data will occur if the hypothesis test is true.
If the hypothesis test is rejected due to a value p≤0.05 the probability that our finding is false positive is 5%.
A p-value=0.05 means the same as a p-value≤0.05.
The p-values are reported as values less than or greater than a nearest value.
Statistical significance is a property of the effect or population under study.
We should always use two-tailed p-values.
When the same hypothesis is tested in different studies, and none or a minority of the tests are statistically significant (p>0.05) then on average the evidence supports the null hypothesis.
When the same hypothesis is tested in two different populations and the p-value results are opposite at the 0.05 threshold, these results are inconsistent.
When the same hypothesis is tested in two different populations and we obtain the same p-values, then the results are concordant.
If we observe a small p-value, there is a good chance that in the next study we will estimate a small p-value for the same hypothesis.

## REFLECTIONS AND CONCLUSIONS

The NHFT and the p-value are widely used in biomedical research; however, there are questions related to their conception, limitations and scope. In academia, it is recognized that misuse and misinterpretation based on its arbitrary categorization constitute a critical element that feeds the replication crisis of science in different disciplines. In this sense, it is crucial to remember that the p-value is calculated from statistical models that have assumptions to be met, which may vary between studies and whose interpretation must be made after assessing the threats to the validity and accuracy of the study.

In view of the above, efforts have been made on several fronts to develop alternatives for analysis and communication of results; both with variations in the thresholds, as well as frequentist (ie: confidence intervals, among others) and Bayesian (ie: Bayes factor, among others) options. Although in this article we do not provide further details or make value judgments regarding the alternatives, we emphasize that in all cases we must interpret the estimates in the light of the inherent strengths and limitations of each technique. We also consider that there is still much work to be done to implement these improvements in a pragmatic and contextualized manner. Finally, beyond the complexity of the analyses and their interpretations, we believe that science is better when we emphasize estimation over testing.
