# Supplementary material for: The null hypothesis significance test and the dichotomization of the p-value: Errare Humanum Est
Source: Rev Peru Med Exp Salud Publica. 2024 Nov 26;41(4):422–30. doi: 10.17843/rpmesp.2024.414.14285. (PMC11797584; doi:10.17843/rpmesp.2024.414.14285.)
Supplement: Translation

# Translation: La prueba de significancia de la hipótesis nula y la dicotomización del valor p: *Errare Humanum Est*

**Authors:** Edward Mezones-Holguín, Ali Al-kassab-Córdova, Percy Soto-Becerra, Sonia Hernández-Díaz, Jay S. Kaufman

**Affiliations:** 1 Centro de Excelencia en Investigaciones Económicas y Sociales en Salud, Universidad San Ignacio de Loyola, Lima, Perú.; 2 Vicerrectorado de Investigación, Universidad Continental, Huancayo, Perú.; 3 Department of Epidemiology, Harvard T. H. Chan School of Public Health, Boston, Massachusetts, EE.UU.; 4 Department of Epidemiology, Biostatistics, & Occupational Health, McGill University, Montreal, Canada.

**Keywords:** Análisis Estadístico, Pruebas de Hipótesis, Bioestadística, Epidemiología y Bioestadística, estadística & datos numéricos.

## Abstract

La toma de decisiones en salud es compleja y requiere informarse en la mejor evidencia científica. En este proceso, la información generada a partir del análisis estadístico de los datos es crucial, el cual puede desarrollarse desde las perspectivas frecuentista o bayesiana. En la arena frecuentista, la prueba de significancia de la hipótesis nula (PSHN) y el *valor p* es una de las técnicas de mayor uso en diferentes disciplinas. No obstante, la PSHN desde la academia ha sido sometida a una serie de cuestionamientos desde diversas aristas, lo cual ha conllevado a situarla como una de las causantes de la denominada *crisis de replicabilidad en la ciencia*. En este artículo de revisión, realizamos un breve recuento histórico sobre su desarrollo, resumimos los métodos subyacentes, describimos algunas controversias y limitaciones, abordamos el mal uso y mala interpretación, para finalmente dar algunos alcances y reflexiones en el contexto de la investigación biomédica.

## INTRODUCCIÓN

En el contexto de la toma de decisiones en salud, la utilización de la evidencia científica es cardinal. La investigación científica generalmente sigue dos enfoques principales: el empírico-inductivo centrado en la generalización a partir de observaciones específicas y el hipotético-deductivo basado en la evaluación de la validez de una hipótesis específica ^(^^1^. En la investigación biomédica, el análisis de datos constituye un desafío tanto para estudios observacionales como para estudios experimentales; donde la validez y la precisión son claves; sin embargo, estas propiedades se ven amenazadas por los errores sistemáticos y aleatorios ^(^^2^. En la práctica, uno de los retos que enfrentamos es el inferir hallazgos en la población de interés en base a una muestra, mediante un procedimiento formal de inferencia estadística con modelos matemáticos que buscan reflejar un fenómeno usualmente complejo ^3^. En el análisis estadístico encontramos dos grandes corrientes: la frecuentista y la bayesiana; ^4^ por lo que resulta crítico comprender las diferencias y similitudes entre ambos enfoques a fin de seleccionar por planificar adecuadamente el diseño de estudio, las técnicas de muestreo y el análisis de datos.

Dentro de las diversas técnicas frecuentistas de inferencia estadística, las de mayor uso son la prueba de significancia de la hipótesis nula (PSHN) y los intervalos de confianza (IC) ^5^^-^^7^. En ambos casos, buscamos responder preguntas acerca de poblaciones basadas en una muestra, con la posibilidad de calcular medidas de asociación multiplicativas o aditivas (i.e., razón de riesgos o diferencia de riesgos, respectivamente), las cuales en ausencia de errores sistemáticos y bajo ciertos supuestos, podrían ser interpretadas como causales (i.e., de efecto) ^2^^,^^8^. Específicamente, la PSHN testea hipótesis en una determinada población de interés mediante la estimación del valor p ^(^^6^^,^^9^.

Más allá de que la PSHN es el enfoque dominante en varias áreas del conocimiento; desde su formulación ha estado sometida a controversia y críticas ^5^^,^^7^^,^^10^^-^^12^. El *valor p* es la probabilidad de obtener un resultado igual al observado, o uno más extremo, si la hipótesis nula es verdadera; y aunque contiene información útil, no representa la magnitud de la asociación evaluada ^6^^,^^7^^,^^9^^,^^13^. Algunos autores han manifestado que la PSHN es una de las técnicas de análisis estadístico con mayor abuso y malas interpretaciones ^7^^,^^10^^,^^11^, situación que han contribuido a la denominada *crisis de la replicabilidad* en investigación científica ^14^^-^^17^. Subsecuentemente, el conocer más acerca de esta métrica en el marco de la investigación biomédica es relevante.

Si bien la PSHN se utiliza en diversos tipos de estudios biomédicos, en este artículo abordamos su uso en estudios primarios (análisis de datos de las unidades primarias de análisis; i.e. estudios observacionales y estudios experimentales) y principalmente para el testeo de asociaciones (puesto que puede utilizarse en otros escenarios, como es el caso de comparación de distribuciones). Primero, presentamos los métodos y realizamos un breve recuento histórico. Segundo, resumimos algunas controversias y limitaciones. Tercero, anotamos algunos malos usos e interpretaciones erróneas. Finalmente, apuntamos sucintamente algunas reflexiones ante la problemática expuesta.

## BASES HISTÓRICAS Y CONCEPTUALES

La PSHN es utilizada para rechazar o no una hipótesis nula en base al rol que puede tener el error aleatorio de muestreo ^2^^,^^18^. La PSHN evoluciona de la combinación de dos orientaciones filosóficas divergentes desarrolladas simultáneamente por Ronald Fisher, y por Jerzy Neyman y Egon Pearson ^9^^,^^13^^,^^19^. 

### La dócima de significancia, test de significancia o probabilidad de significancia

Fue publicada en 1925 por Ronald Fisher, esta técnica evaluaba si el resultado es significativo mediante la *probabilidad de significancia* (PS), una medida de la consistencia entre los datos y la hipótesis nula con valores de 0 a 100% donde a menor valor, mayor consistencia. La PS fue propuesta como una herramienta inferencial que buscaba apartarse del subjetivismo de la orientación bayesiana. Fisher consideraba que esta herramienta debería combinarse con otras fuentes de información y de utilizase un umbral, este debería ser flexible y variar en función del conocimiento acumulado sobre la pregunta de investigación ^13^^,^^19^^,^^20^.

### La dócima de hipótesis o prueba de hipótesis

En 1933, Jerzy Neyman y Egon Pearson propusieron la inclusión de hipótesis alternativa (inicialmente formulada en 1928) y un enfoque teórico que implicó definir y considerar a los errores aleatorios tipo 1 y tipo 2. Buscaban estimar un efecto mínimo relevante basado en la cuantificación de la magnitud del error aleatorio y su ajuste a largo plazo con la utilización de regiones críticas a fin de definir el rechazo o no rechazo de una hipótesis, bajo el supuesto que no podía establecerse conclusiones robustas a partir de un solo estudio ^13^^,^^19^^,^^20^.

Tras años de constantes críticas del pensamiento entre ambas escuelas, hacia 1940, otros investigadores -entre ellos *Lindquist-* crearon un sistema que recogió ambas aproximaciones; al cual le denominaron: *dócimas de hipótesis basadas en el valor p*, *dócimas de significancia estadística* o *prueba de significancia de la hipótesis nula*^13^. En esta propuesta excluyeron algunos puntos relacionados a lo formulado por Fisher (en cuanto a la paráfrasis de la incorporación del conocimiento acumulado) y por *Neyman y Pearson* (que permite interpretar como limitada la conclusión derivada de un único experimento) ^(^^9^^,^^13^^,^^19^^,^^21^. Precisamente la conceptualización de la PSHN a partir de dos enfoques con métodos y terminologías diferenciadas han contribuido al desarrollo de controversias en la academia ^9^^,^^13^^,^^21^.

Para entender la PSHN, debemos conocer acerca de los errores aleatorios de muestreo: el error tipo 1 alude al falso positivo de rechazar una hipótesis nula verdadera; mientras que el error tipo 2, corresponde al falso negativo de no rechazar la hipótesis nula cuando es falsa. Bajo la perspectiva frecuentista, podemos controlar este error con la prefijación de la probabilidad de ocurrencia de estos errores si se tomaran infinitas muestras posibles del mismo tamaño. A la probabilidad del error tipo l (α) prefijada en el diseño le conocemos como significancia y a su complemento (1-α), confianza. Mientras que, la probabilidad del error tipo 2 es denotada como β y su complemento (1-β) como potencia estadística ^2^^,^^18^. Para la ilustrar estos conceptos, en la Figura 1 presentamos la distribución de probabilidades de los estadísticos muestrales de prueba que obtendríamos aleatoriamente en dos escenarios posibles: si la hipótesis nula es verdadera o si la hipótesis alternativa es verdadera para un tamaño de efecto determinado.

**Figura 1:** Distribución de probabilidades de los estadísticos muestrales de prueba obtenidos aleatoriamente en escenarios de hipótesis nula o alterna verdadera.

Usualmente ambas probabilidades las establecemos durante el cálculo del tamaño de muestra, donde predefinimos el α y tras la ejecución de la prueba, estimamos el *valor p*; es en función del contraste entre el *valor* p y el α, que rechazamos o no la hipótesis nula y así definimos el rechazo en base a la magnitud de la incompatibilidad entre los datos observados y la hipótesis nula ^6^^,^^9^^,^^13^^).^ En un contexto donde suelen usarse de manera indistinta, resulta crucial que diferenciemos el valor p de la PSHN; de manera general, la PSHN corresponde al proceso de testeo y el *valor p* su principal indicador ^2^^,^^9^^,^^18^.

La PSHN corresponde a la especificación de una hipótesis nula acerca de parámetros poblacionales, donde manifestamos la no existencia de asociación o de diferencias expresada en escala aditiva o multiplicativa en un modelo estadístico. En adición, consideramos hipótesis alternativas (dócimas) que presentan asociación o diferencias a una o a dos colas; esta última es la de uso más frecuente y testea la existencia de asociación o diferencias independientemente de su sentido; en cambio, en la dócima a una cola la asociación o diferencia se prueba a favor de uno de los sentidos ^6^^,^^13^^,^^18^.

En el proceso de la PSHN, calculamos el estadístico de prueba observado para un modelo en específico y a partir de la distribución esperada del estadístico si la hipótesis nula fuese verdadera, estimamos un *valor p*, el cual resulta ser la probabilidad de obtener un estadístico de prueba igual o más grande que el observado en nuestra muestra si repitiésemos el muestreo infinitas veces más bajo una hipótesis nula verdadera. Así, el *valor p* nos dice que cuando no hay efecto (efecto nulo), no hay asociación o no hay diferencia, es posible ver estimaciones muestrales de efecto diferentes a cero (i.e., valores como 1, 2, o 10 mm Hg de diferencias de presión arterial) simplemente por azar y nos cuantifica que tan probable es observar estas diferencias o diferencias más extremas si realmente no existiesen esas diferencias en la población. Así, si un *valor p* es *muy pequeño ”*implica que, aunque posible, es muy poco probable haber obtenido un estadístico de prueba igual o más grande que el observado de una hipótesis nula verdadera. En este escenario, asumir que la hipótesis nula es cierta sería reconocer que es más probable que suceda lo improbable, por tal motivo, lo más razonable y usual es usar la regla del suceso infrecuente, donde consideramos que la hipótesis nula no puede ser cierta y rechazarla, con lo cual optamos por la alternativa ^(^^6^^,^^9^^,^^18^^,^^22^. Adicionalmente, el no poder rechazar la hipótesis nula no es equivalente a confirmarla y es este un punto crítico en la construcción de las conclusiones ^(^^7^^,^^13^^,^^18^.

En la Figura 2, mostramos la curva que denota la probabilidad bajo la hipótesis nula de todos los valores posibles del estadístico de prueba de un estudio en particular tanto a una cola como a dos colas. En el lado derecho observamos el área correspondiente a α y al *valor p*. Si bien son semejantes, la diferencia clave recae en que a α lo preespecificamos, mientras que el *valor p* lo calculamos a partir de los datos observados en el estudio.

**Figura 2:** Curva de probabilidad bajo la hipótesis nula de todos los valores posibles del estadístico de prueba con una y dos colas.

Volvemos a enfatizar que, el *valor p* solo toma en cuenta el error aleatorio, por lo que su interpretación nominal debe tomarse exclusivamente en ausencia de otros errores en el estudio, tales como, sesgo de selección, sesgo de medición, confusión, o error en la especificación del modelo ^(^^2^^,^^7^^,^^22^. En ese sentido, el adolecer de falta de asignación aleatoria en los estudios observacionales o la violación de ésta en estudios experimentales trae consigo un problema mayor que la PSHN, al afectar a la validez de los resultados ^2^^,^^5^^,^^22^^,^^23^.

## CONTROVERSIAS Y LIMITACIONES

En esta sección abordamos algunos aspectos críticos en relación con la PSHN y el *valor p*, si bien los presentamos de manera independiente, en la práctica estas pueden superponerse e interactuar.

### Problemas en la concepción

Más allá de lo polémico de su creación a partir de dos corrientes estadísticas contrapuestas, se ha argumentado que la PSHN y el *valor p* tienen *problemas en su concepción misma*. La PSHN se basa en evaluar la probabilidad de obtener los datos observados bajo la suposición de que una hipótesis nula específica es verdadera [Pr (datos observados | H_0_ verdadera)]. Sin embargo, lo que realmente nos interesa es calcular la probabilidad de que la hipótesis nula sea verdadera dada la evidencia recolectada [Pr (H_0_verdadera|datos)]. Es así como, para hacer este salto lógico, ejecutamos un procedimiento reconocido en epistemología como *deducción inversa* o *método de reducción,* cuando el hallazgo observado es improbable para obtener una conclusión acerca de la probabilidad de la hipótesis nula dada la conclusión observada en los datos, para lo cual requerimos asumir varios supuestos ^24^^-^^26^. Asimismo, se esgrime que, bajo el enfoque frecuentista, resulta difícil atravesar la brecha lógica desde la probabilidad del hallazgo y de hallazgos más extremos, dada cierta hipótesis nula, hacia una decisión sobre si se debe aceptar o rechazar dicha hipótesis ^9^^,^^12^^,^^14^^,^^17^^,^^25^^,^^26^. Consecuentemente, la interpretación de los resultados basados en la PSHN y el *valor p* requiere que conozcamos plenamente los supuestos subyacentes al proceso.

### Dependencia del tamaño de muestra y discordancia con el tamaño de efecto

Dado que en la PSHN requerimos una especificación a priori de la probabilidad del error tipo 1, ello tiene una implicación directa sobre el tamaño de muestra calculado y sobre el *valor p* aceptado para la definición de significancia ^6^^,^^13^^,^^18^. En muestras grandes, incluso si el efecto fuera mínimo, el *valor p* podría ser extremadamente pequeño, lo que facilita el rechazo de la hipótesis nula, independientemente del tamaño del error tipo 1 prefijado. Por tanto, el *valor p* podría llegar a ser tan pequeño como lo permita el tamaño de la muestra, lo cual hace que el análisis sea susceptible a manipulación ^10^^-^^12^. Por ejemplo, en el caso de análisis con datos masivos (*big-data*), la estimación del *valor p* podría prácticamente definir cualquier *asociación* como *estadísticamente significativa*^9^^,^^11^^,^^23^.

En este sentido, aunque el *valor p* es función del tamaño de la muestra y nos provee la probabilidad de observar el estadístico de prueba estimado, este estadístico no nos brinda información sobre la magnitud del efecto observado. Al tratarse de una métrica compuesta que depende en gran medida del tamaño de la muestra, implica que pequeñas diferencias en el efecto en un número suficientemente grande de observaciones pueden cobrar significancia estadística; por lo contrario, diferencias mayores de efecto en un número reducido de observaciones pueden no alcanzarla ^7^^,^^10^^,^^23^^,^^27^^,^^28^. Es así, que en los ensayos clínicos se ha estimado que la *significancia estadística* tiende a sobreestimar seriamente el efecto del tratamiento y que algunos resultados *no significativos* corresponden a importantes efectos ^29^^,^^30^.

### No correspondencia con la importancia clínica

La significancia estadística no es equivalente a la importancia científica, humana, económica o clínica ^23^^,^^29^^,^^30^^,^^33^^,^^34^. Reiteramos que la definición formal del *valor p* es un concepto matemático expresado acerca de una probabilidad condicionada sobre la nulidad, por lo que es factible que existan diferencias estadísticamente significativas pero clínicamente no relevantes, o lo inverso ^6^^,^^7^. Se reconoce que las diferencias clínicas son más importante que las estadísticas, es así como, la evaluación del *valor p* es secundario en la gradación de la calidad de la evidencia y no es una medida de evidencia en sí misma ^(^^23^^,^^35^.

Para ilustrar lo expuesto, en la Figura 3, mostramos un ejemplo con la presión arterial para un escenario clínicamente irrelevante (reducción de 1 mm Hg) y un escenario clínicamente significativo (reducción de 10 mm Hg). Para un mismo valor de tamaño de efecto y α preespecificado, el *valor p* cambia conforme el tamaño de muestra se incrementa (ambos tamaños de efecto son *estadísticamente significativos* conforme mayor es el tamaño de muestra); sin embargo, las implicaciones clínicas son distintas.

**Figura 3:** Escenarios clínicamente irrelevantes o estadísticamente significativos a propósito de ejemplo en los cambios en la hipertensión arterial

### Categorización/dicotomización arbitraria

El *valor p* es una medida numérica continua con valores que están entre 0 y 1; sin embargo, lo más frecuente es interpretarlos de la forma binaria: *estadísticamente significativo* y *no estadísticamente significativo,* basados en un umbral arbitrario que usualmente corresponde a 0,05 de alguna literatura por algunos reportes considera el *valor p* menor a 0,01 como *muy significativo*^9^^-^^11^. En la literatura biomédica se ha estimado (basados en artículos indexados en Medline) que el 96% de los resúmenes y artículos a texto completo reportan *valores p* de 0,05 o menores ^5^. Aunque en otras disciplinas se ha clasificado adicionalmente en: altamente significativo, marginalmente significativo y estadísticamente no significativo; con puntos de corte en 0,01 y 0,1^6^. Debemos enfatizar que, la categorización conlleva a pérdida de información relevante sobre el parámetro de interés ^7^^,^^11^^,^^36^. En base a ello, algunos investigadores sostienen que si utilizamos el *valor p* debería presentarse en su naturaleza continua y siempre debe concluirse bajo un contexto específico^29^^,^^37^.

### Pobre replicabilidad, la maldición del ganador y vicios derivados de la búsqueda de lo *estadísticamente significativo*

La confiabilidad de la PSHN y el *valor p* ha sido puesta en duda, debido a la alta frecuencia de hallazgos científicos positivos *estadísticamente significativos*, los cuales se contradicen en estudios subsecuentes o en experimentos repetidos ^14^^-^^16^^,^^38^^-^^40^. Es precisamente, la combinación de la dependencia del tamaño de muestra y la dicotomización arbitraria las que - entre otros factores - han traído consigo críticas por su pobre replicabilidad y alta proporción de falsos positivos ^28^^,^^30^^,^^33^^,^^39^.

Se ha descrito que, bajo el umbral de 0,05 para el *valor p*, se produce un riesgo de hallazgos falsos positivos de 13% en ensayos clínicos publicados en revistas indizadas a Pubmed-Medline ^(^^41^, asimismo, se han observado discordancias en otras series de ensayos clínicos analizados ^42^. Además, basado en datos del *Open Science Colaboration*, se calculó un coeficiente de correlación de 0,004 (bajo) entre los *valores p* obtenidos de la cohorte original de estudio y aquellos estimados a partir de las cohortes de replicación ^28^. Nueve de cada diez ensayos clínicos no alcanzan una potencia estadística del 80% (mediana:13%) y la mayoría no podrían abordar el efecto de la intervención ^31^. Esto implica que, si se produce evidencia con resultados estadísticamente significativos con una potencia insuficiente, el tamaño del efecto podría ser exagerado, por lo cual no sería replicable en futuros estudios y llevaría a interpretaciones erróneas. Este fenómeno se conoce como la *maldición del ganador* y se acuñó a partir de la idea de una subasta donde se está adivinando el verdadero valor del artículo subastado. El ganador de la subasta es el que paga el mayor precio para competir con otros potenciales compradores que pujan por el artículo. Aunque el promedio de todas las pujas es no sesgado, el precio pagado al final por el ganador definitivamente es el que sobreestima en mayor magnitud el verdadero valor del objeto. De esta manera, el investigador con el resultado *estadísticamente significativo* es como el ganador de la subasta: seguramente será el que más se aleje del valor real, a menudo, sobreestimándolo ^31^^,^^32^. Así, el *valor p maldice* al investigador con estimaciones de efecto infladas, pero estadísticamente significativas.

En adición, son varias las malas prácticas de investigación relacionadas a la búsqueda de encontrar y reportar resultados *estadísticamente significativos*, entre las cuales encontramos a la multiplicidad (*multiple comparison*), los reportes selectivos (*Cherry-picking*), las expediciones de pesca (*fishing*), el uso selectivo de los datos (*Data Dredging)*, la piratería (*P-hacking*) y el sesgo de publicación (*publication bias*) ^43^. Cuando conducimos múltiples pruebas estadísticas dentro de un mismo estudio aumenta la probabilidad de observar al menos un resultado *estadísticamente significativo* por azar sin que haya una asociación o efecto real; dado que un artículo típico contiene docenas de pruebas, un porcentaje de ellas podría ser *estadísticamente significativas*, las cuales al ser resaltadas conducen a un error de replicación ^33^^,^^44^^-^^46^. Asimismo, la manipulación en la búsqueda de resultados *significativos* resulta en el análisis selectivo de datos o emplear múltiples pruebas hasta conseguir un desenlace deseado ^47^. En conjunto, estas prácticas derivan en un aumento de la probabilidad de *error tipo 1* y de conclusiones engañosas ^22^.

Ante la creciente ola de criticismo, la Asociación Americana de Estadística (ASA del acrónimo en inglés) realizó un pronunciamiento con seis enunciados claves acerca del *valor p*, los cuales mostramos en su versión traducida al español en la Tabla 1 ^30^. Si bien, ha tenido apreciaciones positivas y negativas en la academia ^48^^,^^49^; a nuestro criterio, la posición de la ASA constituye un esfuerzo válido en el intento de redireccionar la práctica científica y académica.

**Tabla 1:** Principios de la Asociación Americana de Estadística acerca del *valor p* (Tomado de: Wasserstein &Lazar) ^30^ *.*

Principios
Los *valores p* pueden indicar el nivel de incompatibilidad entre los datos observados con respecto a lo preespecificado en un modelo estadístico.
Los valores *p* no miden la probabilidad de que la hipótesis estudiada sea verdadera o la probabilidad de que la información generada a partir de los datos se produzca sólo por el azar.
Las conclusiones científicas, así como las decisiones comerciales, clínicas o políticas no deben basarse únicamente en el hecho de que el *valor p* pase un umbral específico.
Un apropiado proceso de inferencia requiere un reporte completo y transparencia.
El *valor p* o la significancia estadística no es una medida del tamaño del efecto o de la relevancia del resultado.
El *valor p* por sí mismo no proporciona una buena medida de evidencia con respecto a un modelo o hipótesis.

## MAL USO E INTERPRETACIONES ERRÓNEAS

Los cuestionamientos de la PSHN y el *valor p* se extienden hacia su uso incorrecto y a las interpretaciones erradas, lo cual constituye uno de los más serios problemas que afectan a la calidad de la investigación científica en diversas áreas ^12^^,^^15^^,^^16^^,^^37^. La complejidad de su interpretación sumado a la facilidad de calcularlo en los paquetes estadísticos pueden explicar el uso excesivo e inapropiado del *valor p*^22^^,^^36^^,^^38^. Si bien, los clínicos y decisores suelen tener alta confianza en la estimación del *valor p*; su interpretación puede ser contraintuitiva, y generalmente, incorrecta ^30^^,^^34^^,^^35^. Incluso se ha reportado problemas de mala interpretación en profesionales con entrenamiento de postgrado en estadística y epidemiología ^50^. Si bien son múltiples las formas de mala interpretación y cada una requiere un análisis en particular, en la Tabla 2 presentamos las más comunes adaptado a partir de lo expuesto por *Greenland et al* ^(^^7^.

**Tabla 2:** Malas interpretaciones más comunes con respecto a la prueba de significancia de la hipótesis nula y el *valor p (*Adaptado de *Greenland et al.*)^7^

Principios
El *valor p* es la probabilidad de que la hipótesis nula es verdadera.
El *valor p* es la probabilidad de que el azar por sí solo produzca la asociación observada.
Un resultado estadísticamente significativo (p≤0,05) significa que la hipótesis nula es falsa o debería ser rechazada.
Un resultado no estadísticamente significativo (p>0,05) significa que la hipótesis nula es verdadera y no debería ser rechazada.
Un *valor p* grande es evidencia en favor de la hipótesis nula.
Un *valor p* mayor que 0,05 significa que se observó un no efecto o que se demostró la ausencia de un efecto.
La significancia estadística indica científicamente que una relación importante ha sido detectada.
La ausencia de significancia estadística indica que el tamaño del efecto es pequeño.
El *valor p* es la probabilidad de que nuestros datos ocurran si la prueba de hipótesis es verdadera
Si se rechaza la prueba de hipótesis debido a un *valor p*≤0,05 la probabilidad de que nuestro hallazgo sea falso positivo es 5%.
Un *valor p*=0,05 significa lo mismo que un *valor p*≤0,05.
Los *valores p* son reportados como valores menores o mayores a un valor más cercano.
La significancia estadística es una propiedad del efecto o población bajo estudio.
Siempre debemos usar *valores p* a dos colas.
Cuando una misma hipótesis es testeada en diferentes estudios, y ninguno o la minoría de las pruebas son estadísticamente significativas (p>0,05) entonces en promedio la evidencia apoya a la hipótesis nula.
Cuando la misma hipótesis es testeada en dos poblaciones diferentes y los resultados del *valor p* son opuestos en función al umbral del 0,05, estos resultados son inconsistentes.
Cuando la misma hipótesis es testeada en dos poblaciones diferentes y obtenemos los mismos *valores p*, entonces los resultados son concordantes.
Si observamos un *valor p* pequeño, hay una buena probabilidad de que en el siguiente estudio estimemos un *valor p* pequeño para la misma hipótesis.

## REFLEXIONES Y CONCLUSIONES

La *PSHN* y el *valor p* son de amplio uso en la investigación biomédica; sin embargo, tienen cuestionamientos relacionados a su concepción, limitaciones y alcances. En la academia, se reconoce que el mal uso e interpretación errónea basados en su categorización arbitraria constituyen un elemento crítico que alimenta *la crisis de replicación de la ciencia* en diferentes disciplinas. En ese sentido, resulta crucial el recordar que el *valor p* se calcula a partir de modelos estadísticos que tienen supuestos que cumplir, los cuales pueden variar entre los estudios y cuya interpretación debe hacerse previa valoración de las amenazas a la validez y precisión del estudio.

En virtud de lo expuesto, desde diversos frentes se han desplegado esfuerzos para desarrollar alternativas de análisis y de comunicación de resultados; tanto con variaciones en los umbrales, así como, opciones frecuentistas (ie: intervalos de confianza, entre otras) y bayesianas (ie: factor de bayes, entre otras). Si bien en este artículo no brindamos mayores detalles ni hacemos juicios de valor frente a las alternativas; remarcamos que en todos los casos debemos interpretar las estimaciones a la luz de las fortalezas y limitaciones inherentes de cada técnica. Consideramos además que, hay aún mucho trabajo por hacer para implementar estas mejoras de manera pragmática y contextualizada. Finalmente, más allá de la complejidad de los análisis y sus interpretaciones, consideramos que la ciencia es mejor cuando enfatizamos la estimación por encima de las pruebas.
